# Localized Muscular Fatigue in Robotic-Assisted Laparoscopic Surgery: Predictive Modeling Study

**DOI:** 10.2196/68536

**Published:** 2025-12-10

**Authors:** Daniel Caballero, Manuel J Pérez-Salazar, Juan A Sánchez-Margallo, Francisco M Sánchez-Margallo

**Affiliations:** 1 Bioengineering and Health Technologies Unit Jesús Usón Minimally Invasive Surgery Centre Cáceres, Cáceres Spain; 2 Scientific Direction Jesús Usón Minimally Invasive Surgery Centre Cáceres, Cáceres Spain

**Keywords:** laparoscopic surgery, robotic-assisted surgery, artificial intelligence, predictive techniques, electromyography, localized muscle fatigue

## Abstract

**Background:**

Robotic-assisted surgery (RAS) has grown rapidly in recent decades, and several RAS procedures have become the standard. However, the physical and mental demands of minimally invasive surgery (MIS) techniques can lead to ergonomic shortcomings for surgeons. Advances in wearable technology and artificial intelligence favor the development of innovative solutions to analyze and improve ergonomic conditions during surgical practice.

**Objective:**

The main objective is the development and validation of a predictive model of localized muscle fatigue from electromyography (EMG) data during conventional laparoscopic surgery (LAP) and RAS.

**Methods:**

Four different tasks were performed on LAP and RAS: dissection, labyrinth, peg transfer, and suturing. A wireless EMG sensor system was used to record muscle activity. Joint analysis of the spectrum and analysis graphs was used to evaluate the localized muscle fatigue. A dataset was generated for each task as a function of surgeons’ expertise level and surgical type. Each dataset was scaled as preprocessing and divided into 2 datasets: 80% for training and 20% for testing. Multiple linear regression (MLR) and multilayer perceptron (MLP) were applied as predictive techniques and validated on all test datasets. *R*^2^ coefficient and root-mean-square error were used to measure the accuracy of the models.

**Results:**

RAS showed less muscle fatigue for novice surgeons compared to LAP practice, although it was higher for expert surgeons. The predictive model achieved satisfactory *R*^2^ and root-mean-square error coefficients for all parameters extracted from the EMG signal, predicting with high accuracy localized muscle fatigue values. The MLR predictive model demonstrated superior performance relative to the MLP model.

**Conclusions:**

Wearable technology and artificial intelligence techniques have been successfully applied for the development and validation of a novel predictive model based on MLR and MLP to predict localized muscle fatigue in MIS.

## Introduction

### Background

Apart from the well-known advantages of conventional laparoscopic surgery (LAP) and robotic-assisted surgery (RAS) for the patient, these techniques have a steep learning curve and are physically and mentally demanding for surgeons, as well as presenting ergonomic challenges. Consequently, this mental and physical burden, combined with potential ergonomic deficiencies in the working environment, can lead to musculoskeletal problems in surgeons [1]. Although ergonomic conditions are considered to have improved in RAS compared to LAP, mainly for the primary surgeon operating from the console, scientific evidence remains scarce [2]. Therefore, further ergonomic analysis studies are needed to thoroughly investigate the ergonomic conditions of the surgeon during RAS, identify potential deficiencies, and develop possible solutions and recommendations.

Minimally invasive surgery (MIS) and, in particular, RAS have grown rapidly in recent decades, and several robotic-assisted procedures have become standard surgical techniques [3]. The advantages of MIS and RAS for patients are widely known, including reduced tissue trauma, better cosmetic outcomes, and reduced hospital stay [4]. However, these techniques present some limitations for the surgeon, such as possible ergonomic deficiencies during long surgeries, or high levels of stress in certain procedures [5], which can be detrimental to the surgeon’s health and have an impact on the quality of surgical procedures and patient care [6].

Surgical robotics offers greater surgical precision, better visual perception by allowing 3D images to be obtained compared to the 2D images of LAP, and better ergonomics for the surgeon, who can operate in a seated position. There are some traditional methods to assess the workload, both mental and physical, of surgeons during their surgical activity, mainly oriented to subjective evaluations such as, for example, the SURG-TLX scales [7]. However, the evolution and miniaturization of sensors have allowed the increasing incorporation of wearable technology in ergonomic and physiological analysis in the surgical setting, which has made it possible to offer objective solutions without interrupting the surgeons’ surgical practice. Some of these objective parameters are the surgeon’s posture through body movement techniques, force/torque analysis [8], muscle activity through electromyography (EMG) signal analysis [9], and stress level through electrocardiogram examination or electrodermal activity (EDA) signals [10].

The application of artificial intelligence (AI) has grown exponentially in its use and development. These algorithms are based on nontrivial processes to discover potentially useful knowledge initially hidden in the data [11,12]. Among the different AI techniques, there are several algorithms that allow the development of predictive models [13,14]. Thus, the use of these predictive models in the field of ergonomics during surgical practice offers us a wide range of possibilities to predict those risk situations related to the surgeon’s health, for example, inadequate posture, the appearance of muscle fatigue, or high levels of stress. Being able to predict and, therefore, reduce these risk situations would potentially improve the health of surgeons and, consequently, the quality of surgical practice.

### Related Works

Several studies have been presented focusing on the ergonomic analysis in RAS. Some of these studies use subjective surveys [15], demonstrating that 56.1% of regularly practicing robotic surgeons continue to experience related physical symptoms or discomfort, including neck stiffness, finger and eye fatigue, among the most common.

Other studies analyzed the surgeon’s posture at the console during surgical practice using traditional photogrammetry methodologies [16]. Ergonomically risky positioning of the neck and elbow was observed in medical students, and robotic experts showed risky positioning in the knee and hip region. Another study objectively assessed ergonomics in surgical robotics using rapid entire body assessment and the rapid upper limb assessment to quantify ergonomic effectiveness [17]. An Xbox Connect camera was used to record the surgeon’s posture at the console. The results indicated a medium risk, with the recommendation that measures need to be taken to improve surgeon ergonomics. However, these photogrammetry-based methods are prone to occlusion problems in such cumbersome environments as operating rooms.

In order to deal with the above limitations, wearable systems such as inertial measurement unit–based systems are used. These systems continuously track the surgeon’s movements without interfering with the sterile environment. In a study during the performance of robotic prostatectomies, it was concluded that the console can limit postures, causing static loads that have been associated with musculoskeletal symptoms for the surgeon’s neck, torso, and shoulders [18].

Other studies have objectively analyzed the surgeon’s muscle loading using surface EMG systems [9]. The results indicated that laparoscopic practice presented more muscle fatigue in the forearm compared to robotic-assisted laparoscopic surgery. Other studies analyzed physiological parameters to examine the surgeon’s health, such as heart rate as a possible indicator of stress [16]. The highest level of stress was obtained in surgical residents. In previous work, we used different wearable technologies to record the posture, muscle activity, EDA, and electrocardiographic signal of surgeons during robotic-assisted surgical practice [19]. The results indicated that robot-assisted procedures showed better ergonomic outcomes for the surgeon compared to LAP.

Regarding the use of predictive models in ergonomics in MIS based on AI techniques, in previous studies, we developed predictive models to predict stress from ergonomic (kinematic, body posture, and angular rate) and physiological (EDA, blood pressure, and body temperature) parameters of the surgeon [5] during RAS procedures. The linear models (multiple linear regression [MLR]) proposed in this study were successfully validated, improving the results achieved by the nonlinear models (support vector machine, multilayer perceptron [MLP]), demonstrating the possibility of predicting factors that help us to improve the surgeon’s health during robotic surgery.

Other studies analyzed and compared the surgeon’s posture between RAS and LAP techniques using motion analysis technology with inertial sensors and developed a predictive model of ergonomically inappropriate postures for the surgeon [20]. MLR and MLP were compared, with the best results obtained when applying MLR as a predictive technique.

In this study, the predictive model developed would allow the evaluation of localized muscle fatigue for RAS using MLR and MLP. To do this, we will use novel wearable technologies in the surgical field in order to study the surgeon’s localized muscle fatigue and to develop predictive models based on AI that enable predicting situations and factors that lead to increased muscle fatigue in the field of MIS, both in LAP surgery and RAS.

This work represents a significant step in the application of cutting-edge technology for the improvement of ergonomic conditions in the surgical field, with the aim of improving the surgeon’s health during surgery, contributing to the improvement of surgical performance and, consequently, the quality of patient care.

### Objective

The main objective of this study is to develop and validate a predictive model of localized muscle fatigue in surgeons based on EMG sensor data during conventional and robot-assisted laparoscopic surgery, using AI techniques (MLP and MLR) and considering different levels of experience (experienced and novice surgeons). The hypothesis of this study is that it would be possible to predict the localized muscle fatigue in MIS by combining wearable technology and AI techniques, with RAS surgery leading to less muscle fatigue for novice surgeons, who would be less conditioned by the habit of LAP practice.

In addition, this study seeks to take a step forward in the prevention and improvement of ergonomic conditions of surgeons, to seek the improvement of their health and, consequently, the quality of surgery and health care of patients.

## Methods

### Experimental Design

In this study, 8 different surgeons with different levels of experience in LAP performed 4 different surgical tasks by LAP and RAS, all of them on a task-simulator training model during 25 surgical sessions.

For each surgical session, the EMG signal was recorded by using a wireless system (EMG TRIGNO Avanti System, Delsys, Natick, Massachusetts, USA) on 7 muscle groups bilaterally: Brachioradialis, Brachii Triceps, Erector Spinae, Gastrocnemius Medial, Middle Trapezius, Upper Trapezius, and Vastus Lateralis.

From these data, a dataset (140,198 records) was generated, on which a preprocessing technique (scaling) has been applied, and then, 2 datasets were generated: 80% of the data for the training dataset and the cross-validation of 10-folds (112,158 records), and the remaining 20% of the data for the test dataset (28,040 records). Records from all 25 sessions appeared in both datasets.

In this study, 2 predictive AI approaches were applied to the training dataset to generate the predictive models: MLP and MLR.

**Figure 1:**
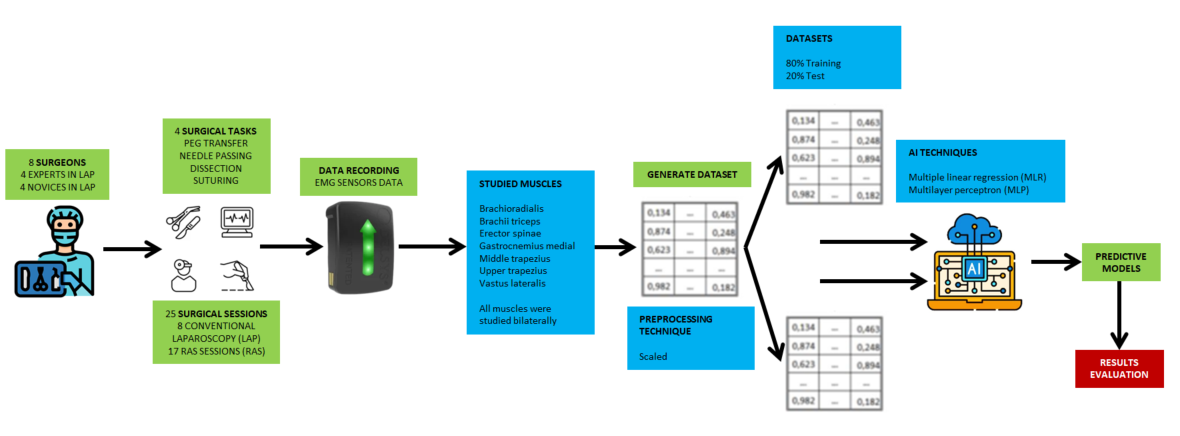
Experimental design. EMG: electromyography; LAP: conventional laparoscopic surgery; RAS: robotic-assisted surgery.

Finally, to validate the generated predictive models, they were validated on the 10-fold cross-validation and test dataset. Figure 1 shows the experimental design followed by this study.

### Experimental Setup

#### Overview

In this study, 4 different surgical activities were performed both by LAP and RAS on a task-simulator training model. For LAP (conventional laparoscopic) practice, the Olympus Visera III System (Shinjuku, Tokyo, Japan) and Karl Storz instruments (Karl Storz SE & Co. KG, Tuttlingen, Germany) were used. For RAS (robotic-assisted laparoscopic surgery), the Versius Surgical System (CMR Surgical, Cambridge, United Kingdom) was used. It is a modular robotic platform with an open console and 3D vision.

In this study, 4 different laparoscopic tasks were performed.

#### Peg Transfer (Hand-Eye Coordination)

**Figure 2:**
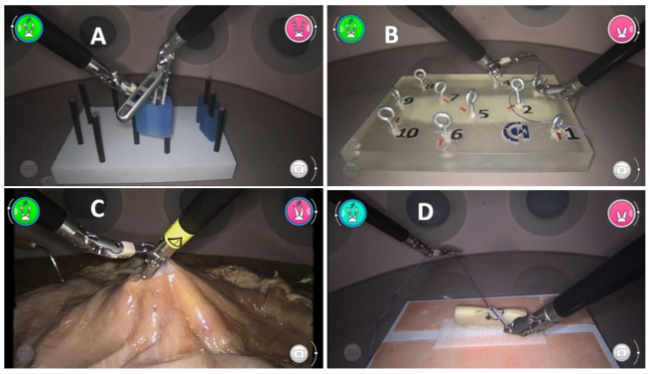
Simulator tasks performed in this study: (A) peg transfer (hand-eye coordination), (B) labyrinth (needle passing), (C) dissection in organic tissue, and (D) suturing.

This task consists of moving rubber pieces with the shape of an elongated toroid from one point to another point, changing the piece from one hand to another hand [21]. For that, 2 fenestrated forceps or a Maryland dissector and a fenestrated forceps were used (Figure 2A). It is considered a repetition when it takes the 3 pieces to the 3 goal points. Participants were asked to complete 2 repetitions with a limit of 10 minutes.

#### Labyrinth (Needle Passing)

In this task, it is necessary to thread a needle through a circuit with holes, in order and in different directions. This task aims to force uncomfortable postures, especially in the wrists, to evaluate the surgeon’s skill and the ease of returning to a correct posture [22]. It is necessary to insert the needle with the dominant hand and remove it from the other side with the nondominant hand. A needle holder was used in the dominant hand and a Maryland dissector in the nondominant hand (Figure 2B). Participants were asked to complete the entire circuit within 10 minutes.

#### Dissection in Organic Tissue

An ex vivo porcine stomach was used for this task. A 3-cm-long incision was made, and the participant was asked to dissect the serous layer from the muscular layer [23]. A scissor was used in the dominant hand and a Maryland dissector in the nondominant hand (Figure 2C). A time limit of 5 minutes was set for this task.

#### Suturing

Participants were asked to perform a suture on the previously performed dissection. The suture consisted of 2 simple sutures and one double suture in opposite directions [24] (Figure 2D). A needle holder was used in each hand, with a time limit of 10 minutes.

### Ethical Considerations

Written informed consent was taken and signed by all participants for the record and use of data generated in this study. In this informed consent, the participants could select to opt out of the study or to participate.

As this research focused only on recording the data that did not contain identifying information and posed no additional risk to subjects and participants, its activities were deemed exempt from ethics committee review. This study was conducted under the guidelines of the Spanish normative 14/2007, of July 3, on Biomedical research [25], since this study does not involve human biological samples or critical data that allow the identification of the participants, and it is not invasive for the human participants.

### Data Recording

**Figure 3:**
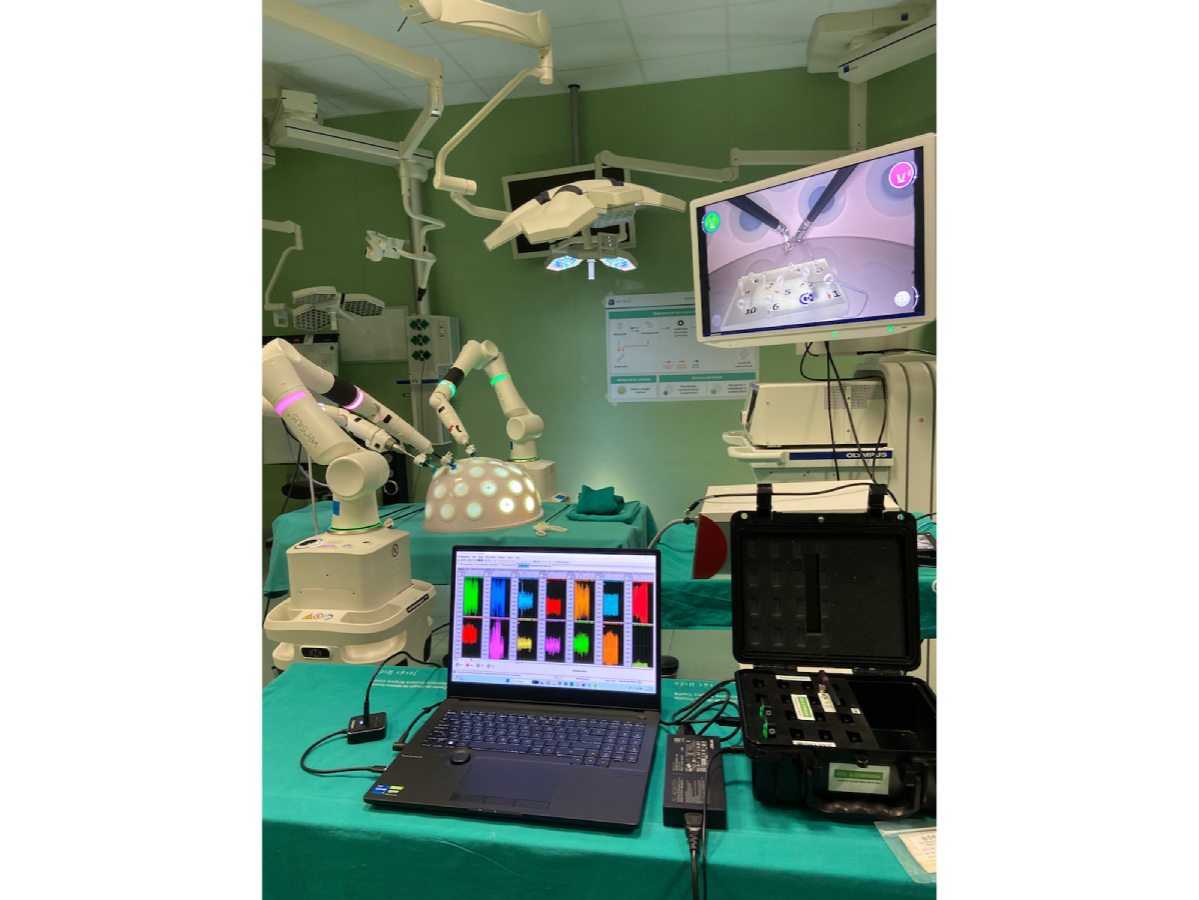
Surgical setting performing labyrinth task on robotic-assisted surgery.

The wireless EMG TRIGNO Avanti System from DELSYS was used to record the muscle activity by means of EMG signals (Figure 3). This system has up to 16 sensors with a sampling rate of 1024 Hz. The EMG signal of the following 7 muscle groups was recorded bilaterally: Brachioradialis, Brachii Triceps, Erector Spinae, Gastrocnemius Medial, Middle Trapezius, Upper Trapezius, and Vastus Lateralis. The EMG sensors were placed on each muscle according to the SENIAM guidelines [26]. These guidelines are widely used in scientific literature, providing detailed recommendations for sensor placement in more than 30 muscles. Among the guidelines, it can be found longitudinal and transverse location, sensor orientation, and interelectrode distance, among others. Prior to the placement of each sensor, the skin was cleaned by gently rubbing it with 70% isopropyl alcohol.

Raw EMG signals were initially processed using a band-pass filter of 20-300 Hz. The filtered EMG was then smoothed with a moving window of 125 milliseconds and calculated as root-mean-square in order to remove artifacts and noise. To normalize the results for each subject, the EMG values were presented as a percentage of maximum voluntary contraction (%MVC). %MVC was computed separately for each muscle group, just before each test, by asking each participant to perform specific contractions against a fixed resistance. The MVC was performed separately for each muscle group just before each test, asking each subject to perform specific contractions against a fixed resistance.

These contractions are performed with the aim of measuring the maximum effort of each muscle group and normalizing the EMG signals of each subject.

### Data Analysis

#### Overview

A total of 8 participants performed the 4 laparoscopic tasks using both LAP and RAS techniques. EMG data were gathered from all sessions. A dataset was generated for each task and as a function of the expertise level of the surgeons and the type of surgery, for a total of 16 datasets. The %MVC was computed for each dataset. Muscular fatigue was quantified using the joint analysis of the spectrum and amplitude method [27].

#### Predictive Model

From all EMG data collected, 16 datasets were generated as a function of the surgical task (peg transfer, needle passing, organic tissue dissection, and suturing), the surgeon’s level of experience (novice and experienced), and the type of surgical technique (LAP and RAS), with 140,198 records. After that, the original datasets were transformed by applying a scaling as a preprocessing technique. The scaled preprocessing technique allows describing each %MVC of each muscle in the scale between 0 and 1 [28]. For this purpose, each value is subtracted by the minimum value and then divided by the interval between the maximum and the minimum values.



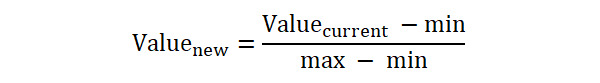



The 16 preprocessed datasets were divided into 32 datasets: 80% of the data for the calibration and training (112,158 records) and 20% for validation and test (28,040 records) purpose [29]. On all calibration and test datasets, data from each participant were added. On all calibration subsets, 2 AI techniques were applied to generate predictive models: MLR and MLP. The free software WEKA (Waikato Environment for Knowledge Analysis; University of Waikato, New Zealand) [30] was used for implementing the predictive models.

MLR is a predictive technique that allows the creation of future models that can be predicted from current data by trend analysis [31]. In this study, MLR was applied as a predictive linear approach on the datasets. MLR was performed, showing the linear relationship between a dependent variable and several independent variables. This technique reaches a linear regression equation that can be used to predict future values.

The M5 method of attribute selection was applied in this study. This method steps through the attributes, removing the one with the smallest standardized coefficient until no improvement in the estimation of the error is observed. In addition, a ridge value of 1.0 × 10^–4^ was applied [32].

MLP was applied as a predictive machine learning approach to datasets. MLPs are a type of artificial neural network model that are developed by using principles of neuronal organization [33]. Thus, different numbers of neurons are aggregated into layers. Different layers may perform different transformations on their inputs. Signals travel from the first layer (the input layer) to the last layer (the output layer), possibly after traversing the layers multiple times [34]. The default configuration was used in this study, with the learning rate equal to 0.3, the number of epochs equal to 500, the threshold equal to 20, and 30 nodes in the first hidden layer, 10 nodes in the second hidden layer, and 3 nodes in the third hidden layer.

Finally, to validate the generated predictive models, they were validated in 2 steps. First, a 10-fold cross-validation, in which the calibration dataset was divided into 10 equally subset, was performed. Each time 1 subset was used to validate, the remaining 9 subsets were used to fit the model. The process was repeated sequentially until all subsets were validated. Therefore, all data were used for both purposes, calibration and validation, making it a very robust method for cross-validation [35]. Finally, the test dataset was used for external validation of the generated predictive models.

The *R*^2^ coefficient was used to evaluate the goodness of fit of the prediction and for its validation, according to the rules given by Colton [36]. This considers *R*^2^ from 0 to 0.25 as a poor to null relationship; from 0.25 to 0.50 indicates a weak degree of relationship; from 0.50 to 0.75 designates a moderate to good relationship; and from 0.75 to 1 shows a very good to excellent relationship.



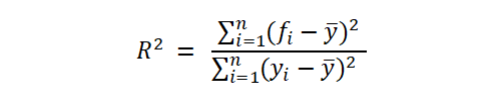



The root-mean-square error (RMSE) was also used to validate the prediction results. The RMSE measures the difference between actual and predicted values. RMSE values less than 0.05 are considered appropriate [37].



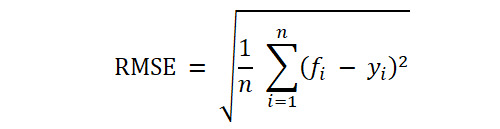



## Results

### Overview

Eight surgeons participated in this study: 4 experts in laparoscopic surgery (> 100 laparoscopic surgeries performed; 1.80, SD 0.08 m tall and 36.5, SD 11.03 years old) and 4 novices in laparoscopic surgery (< 100 laparoscopic surgeries performed; 1.82, SD 0.04 m tall and 28.5, SD 7.59 years old). All participants had no previous experience in robotic-assisted laparoscopic surgery. They performed 8 sessions by means of LAP techniques and 17 sessions using RAS techniques.

The results show the comparison of kinematic data as a function of the surgical technique and the level of expertise of the surgeon, as well as the goodness of the predictive models to predict the localized muscle fatigue for each combination of surgical technique and level of expertise on each muscle group analyzed. These results demonstrate the influence of previous experience in LAP (without previous experience in RAS) on the onset of localized muscle fatigue in robot-assisted LAP practice [38].

### Kinematic Analysis

#### Comparative as a Function of the Surgical Technique

**Figure 4:**
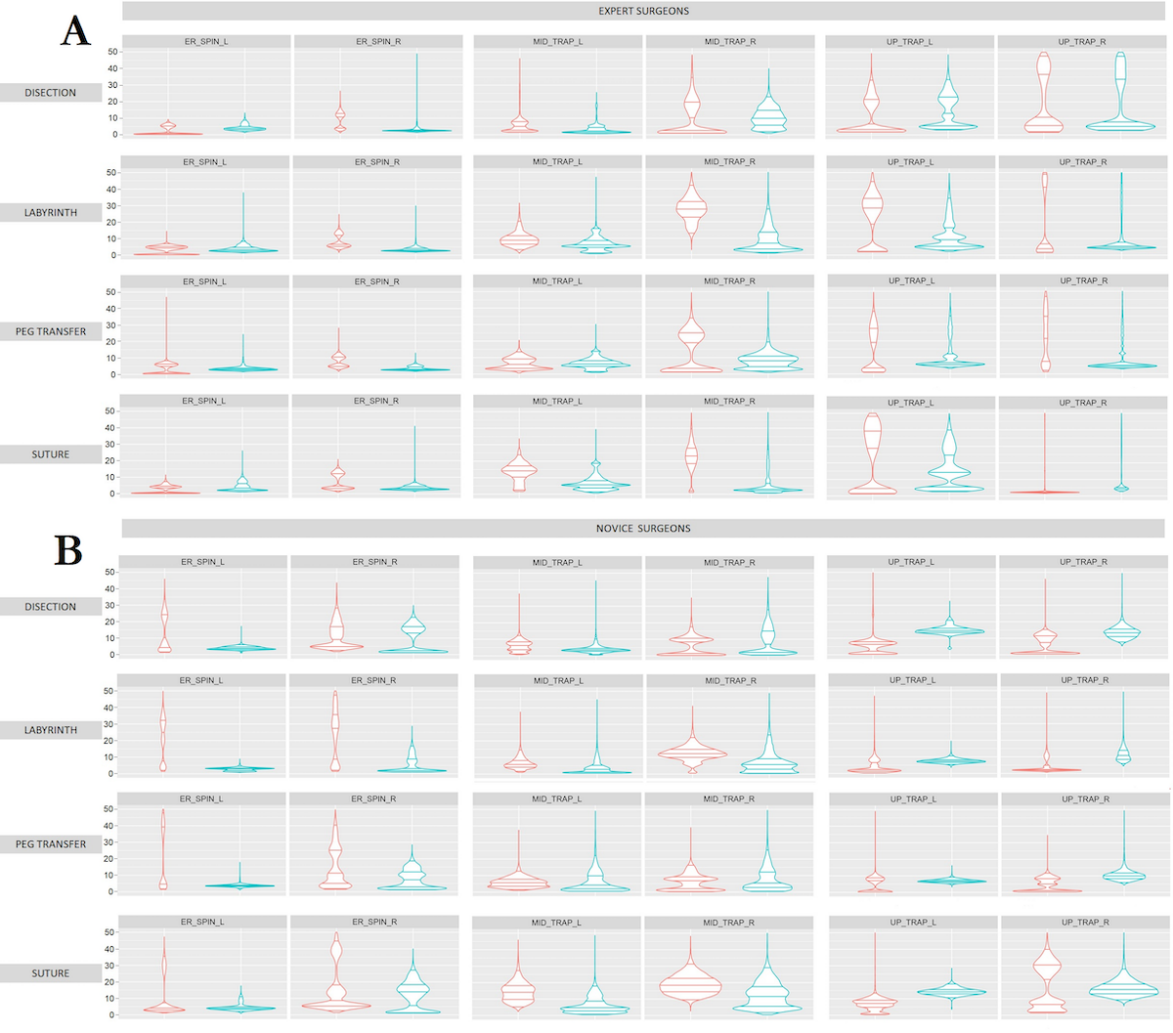
Comparison of muscle activity (percentage of maximum voluntary contraction) of (A) experienced and (B) novice surgeons during performance of 4 simulator tasks, from upper to bottom: dissection, labyrinth, peg transfer, and suturing. Using conventional laparoscopic surgery (red) and robotic-assisted (blue) surgery for the following muscle groups bilaterally: Erector spinae (ER_SPIN), Middle trapezius (MID_TRAP), and Upper trapezius (UP_TRAP).

In general, the erector spinae, middle trapezius, and upper trapezius were the muscles that required the most muscle activity (Figure 4). Similarly, in the case of expert surgeons, for the labyrinth, peg transfer, and suturing tasks, they had lower muscle activity in the muscles on the right side, usually the dominant side, (Figure 4), and for the dissection task, they had lower muscle activity in the muscles on the left side (Figure 4). For the novice surgeons, the peg transfer tasks had lower muscle activity in the muscles on the right side (Figure 4), and for the remaining tasks, lower muscle activity in the muscles on the left side (Figure 4).

**Figure 5:**
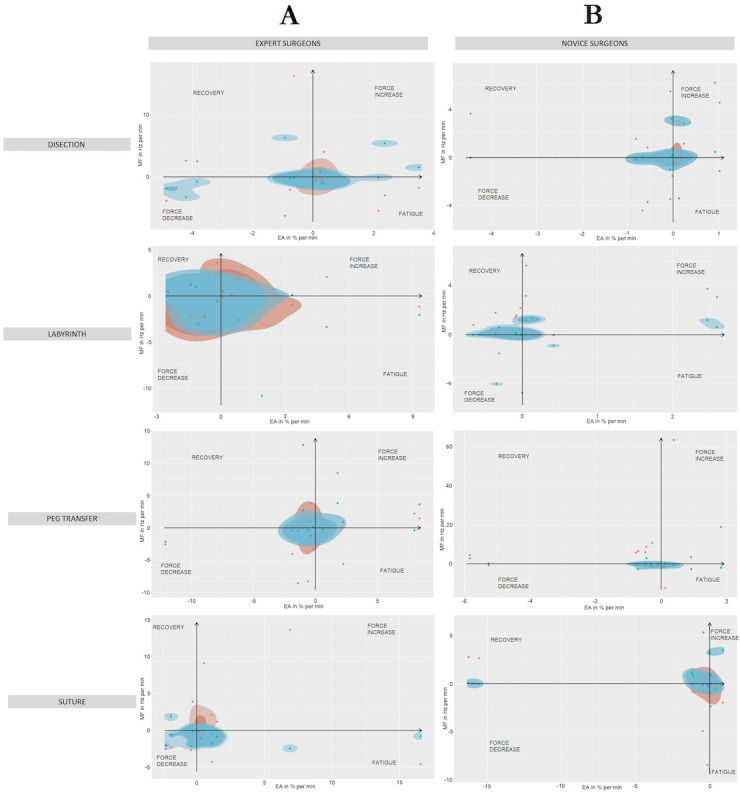
Comparison of fatigue/recovery muscle localized and muscle strength increase/decrease for (A) experienced and (B) novice surgeons on 4 simulator tasks, from upper to bottom: dissection, labyrinth, peg transfer, and suturing. Performing conventional laparoscopic (red) and robotic-assisted (blue) surgeries.

The analysis of localized muscle fatigue showed greater use of force and greater muscle fatigue for RAS in expert surgeons (Figure 5). In the case of novice surgeons, the results were more balanced (Figure 5). However, during LAP, the use of force and localized muscle fatigue is higher, being remarkable for the suturing task (Figure 5).

#### Comparative as a Function of the Level of Expertise

**Figure 6:**
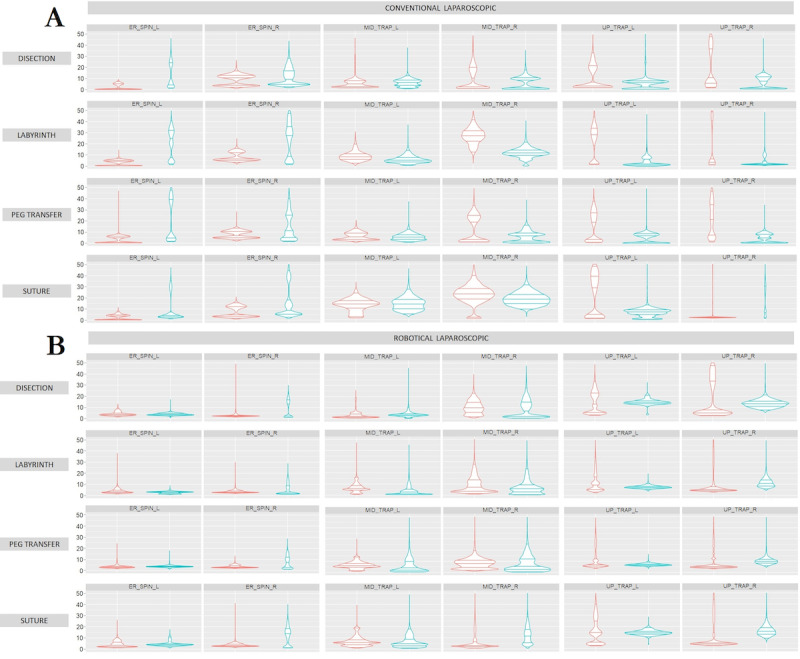
Comparison of muscle activity (percentage of maximum voluntary contraction) of (A) conventional laparoscopic and (B) robotic-assisted surgery during performance of 4 simulator tasks, from upper to bottom: dissection, labyrinth, peg transfer, and suturing. Performing experienced (red) and novice (blue) surgeons for the following muscle groups bilaterally: Erector spinae (ER_SPIN), Middle trapezius (MID_TRAP), and Upper trapezius (UP_TRAP).

Expert surgeons showed greater muscle activity in the forearm muscles (middle trapezius, upper trapezius, triceps brachii, and brachioradialis) than novice surgeons during LAP practice (Figure 6). However, in the rest of the muscle groups, expert surgeons showed less muscle activity. In the case of RAS, in general, novice surgeons showed less muscle activity than expert surgeons (Figure 6).

**Figure 7:**
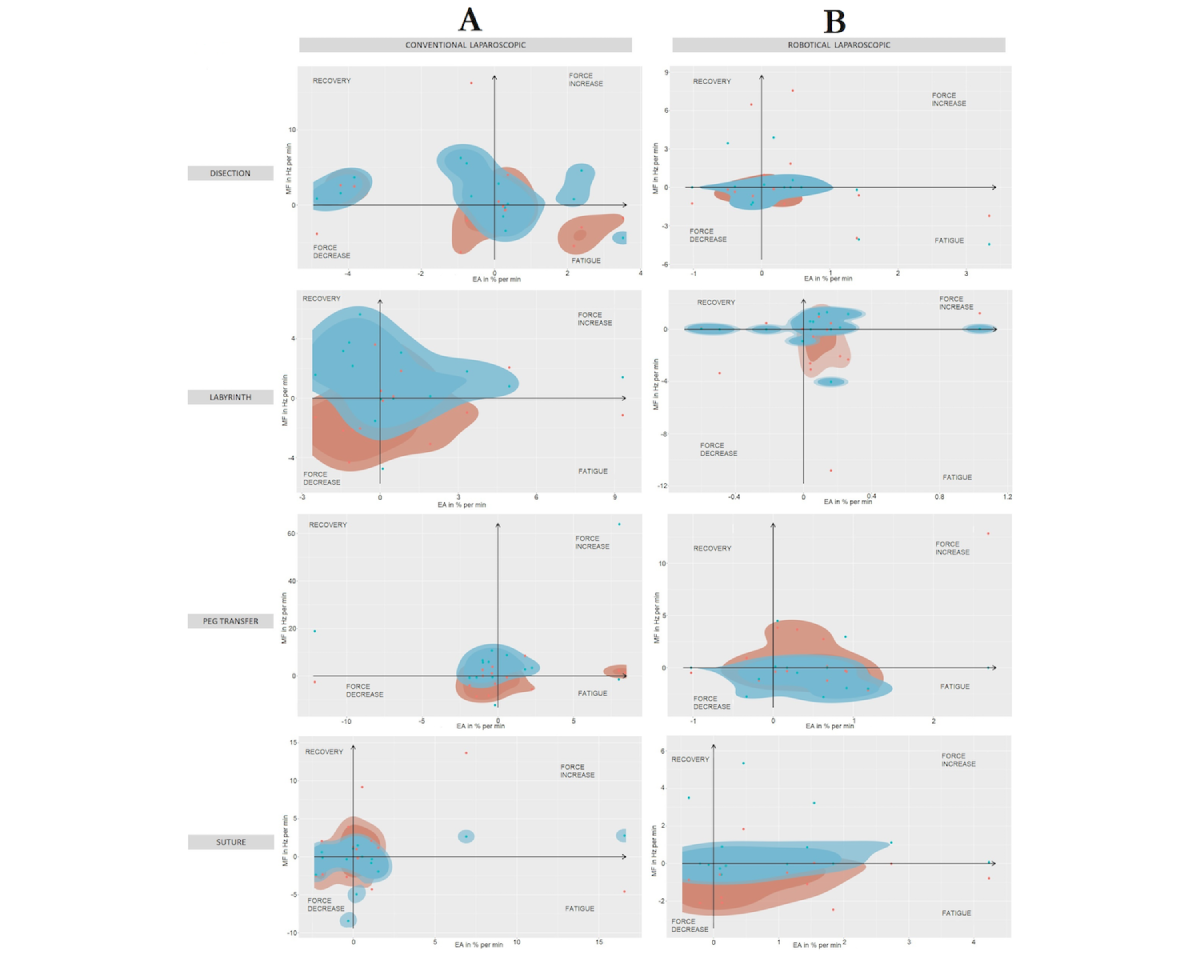
Comparison of fatigue/recovery muscle localized and muscle strength increase/decrease during (A) conventional laparoscopic and (B) robotic-assisted surgeries on 4 simulator tasks, from upper to bottom: dissection, labyrinth, peg transfer, and suturing. Performed by experienced (red) and novice (blue) surgeons.

In general, expert surgeons showed greater localized muscle fatigue and greater use of force during LAP (Figure 7). On the other hand, novice surgeons showed less muscle fatigue, greater use of force, and muscle recovery (Figure 7). In the case of RAS, in general, expert surgeons showed greater use of force and greater localized muscle fatigue (Figure 7). In the case of novice surgeons, they showed less localized muscle fatigue and greater muscle recovery with less use of force (Figure 7).

### Predictive Models

#### Training Dataset

**Figure 8:**
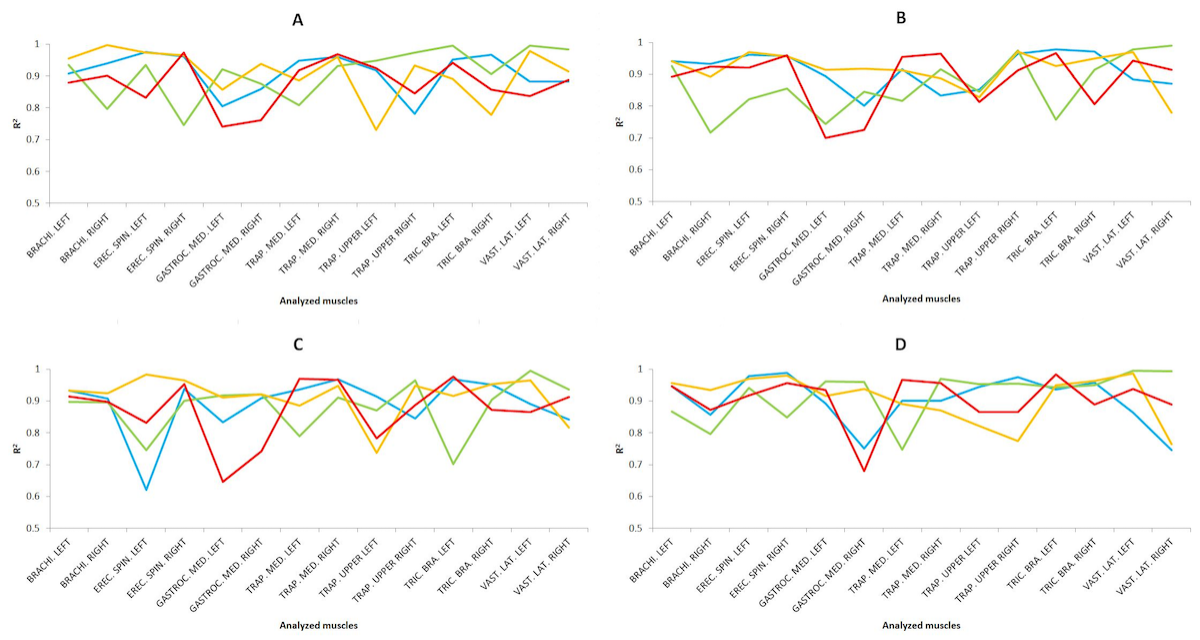
Results from the calibration dataset indicate the R2 values applying the best model in each case on four simulator tasks: (A) dissection, (B) labyrinth, (C) peg transfer, and (D) suturing. For the 4 analyzed groups as a function of the surgical type and the surgeons’ level of expertise, being novice surgeons and conventional laparoscopic surgery (yellow), novice surgeons and robotic-assisted surgery (red), experienced surgeons and conventional laparoscopic surgery (blue), and experienced surgeons and robotic-assisted surgery (green).

The predicted results for the training dataset are shown in Figure 8 for the different tasks. This shows the *R*^2^ coefficient values of the training dataset for the predictive analysis performed applying MLR and MLP, showing the higher of the two in Figure 8, with the dataset preprocessed using the scaling technique. In general, MLR showed slightly higher values than MLP for the *R*^2^ coefficient with a low RMSE error (RMSE < 0.05) for both cases.

In general, the results obtained in the present study are satisfactory according to the standards given by Colton [36]. High to excellent correlations (*R*^2^>0.75), close to 1, and RMSE close to 0 were achieved for all the muscle groups studied. Of note were the *R*^2^ coefficient values during LAP for the right brachioradialis in the dissection task (*R*^2^=0.9963) and the left erector spinae in the pin transfer task (*R*^2^=0.9828) of novice surgeons, and the right erector spinae of expert surgeons in the suturing task (*R*^2^=0.9881). For RAS surgery, right triceps brachii in the dissection task (*R*^2^=0.9953), left vastus lateralis in the dissection (*R*^2^=0.9955), suture (*R*^2^=0.9951), and pin transfer (*R*^2^=0.9949) tasks, and right vastus lateralis in the suture (*R*^2^=0.9933) and labyrinth (*R*^2^=0.9904) tasks of novice surgeons were noteworthy.

#### Prediction Results of the Cross-Validation

**Figure 9:**
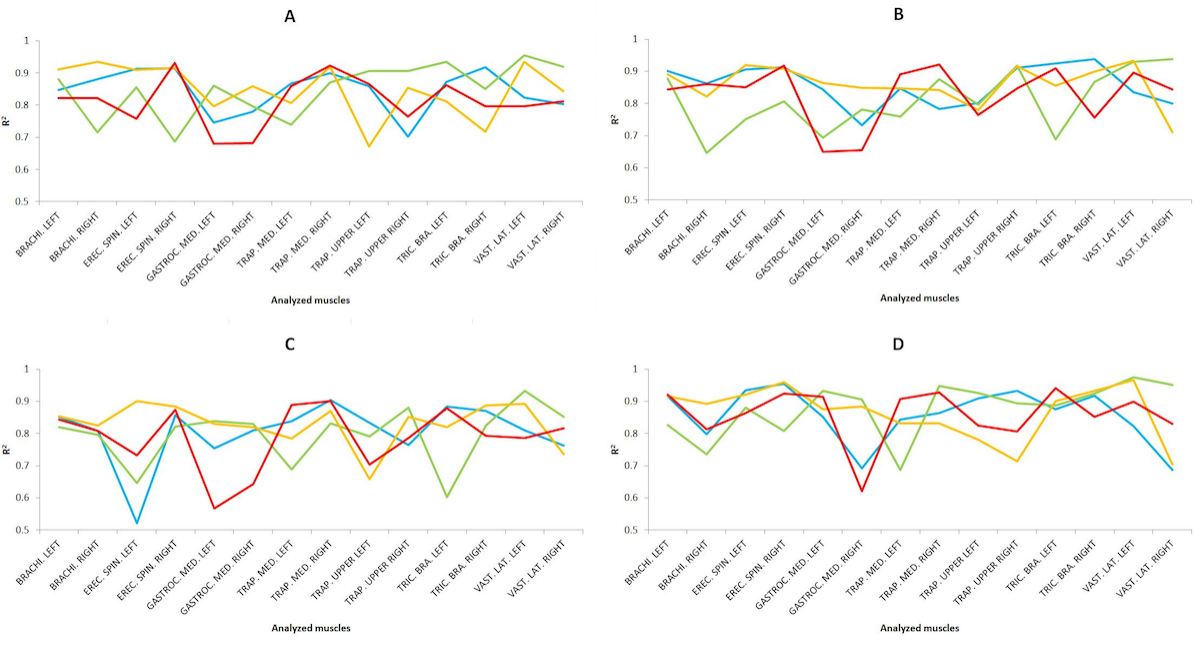
Results from cross-validation indicate the R2 values applying the best model in each case on four simulator tasks: (A) dissection, (B) labyrinth, (C) peg transfer, and (D) suturing. For the 4 analyzed groups as a function of the surgical type and the surgeons’ level of expertise, being novice surgeons and conventional laparoscopic surgery (yellow), novice surgeons and robotic-assisted surgery (red), experienced surgeons and conventional laparoscopic surgery (blue), and experienced surgeons and robotic-assisted surgery (green).

The results obtained from the cross-validation are shown in Figure 9 for each task. Thus, the *R*^2^ coefficient values of the cross-validation for the predictive analysis performed by applying MLR and MLP are shown, with the higher of the two in Figure 9, with the dataset preprocessed using the scaling technique. Overall, MLR showed slightly higher values than MLP for the *R*^2^ coefficient with a low RMSE error (RMSE<0.05) for both cases.

In general, the results obtained in this study are adequate, according to the standards established by Colton [36]. Of note were the *R*^2^ coefficient values for the LAP technique with regard to the right erector spinae during the suturing task (*R*^2^=0.9606) for novice surgeons. Regarding RAS, of note were the right middle trapezius in the suturing task (*R*^2^=0.9472) and the left vastus lateralis in the dissection (*R*^2^=0.9541) and suturing (*R*^2^=0.9746) tasks for expert surgeons, as well as the left triceps brachii (*R*^2^=0.9408) and the right vastus lateralis (*R*^2^=0.9511) in the suturing task for novice surgeons.

In almost all cases, a good to excellent relationship was achieved (*R*^2^>0.6), highlighting that for the dissection task, expert surgeons with RAS achieved the highest *R*^2^ values for most parameters, for the labyrinth task, novice surgeons with LAP, for the peg transfer task, novice surgeons with LAP, and for the suturing task, expert surgeons with RAS.

#### Test Dataset

**Figure 10:**
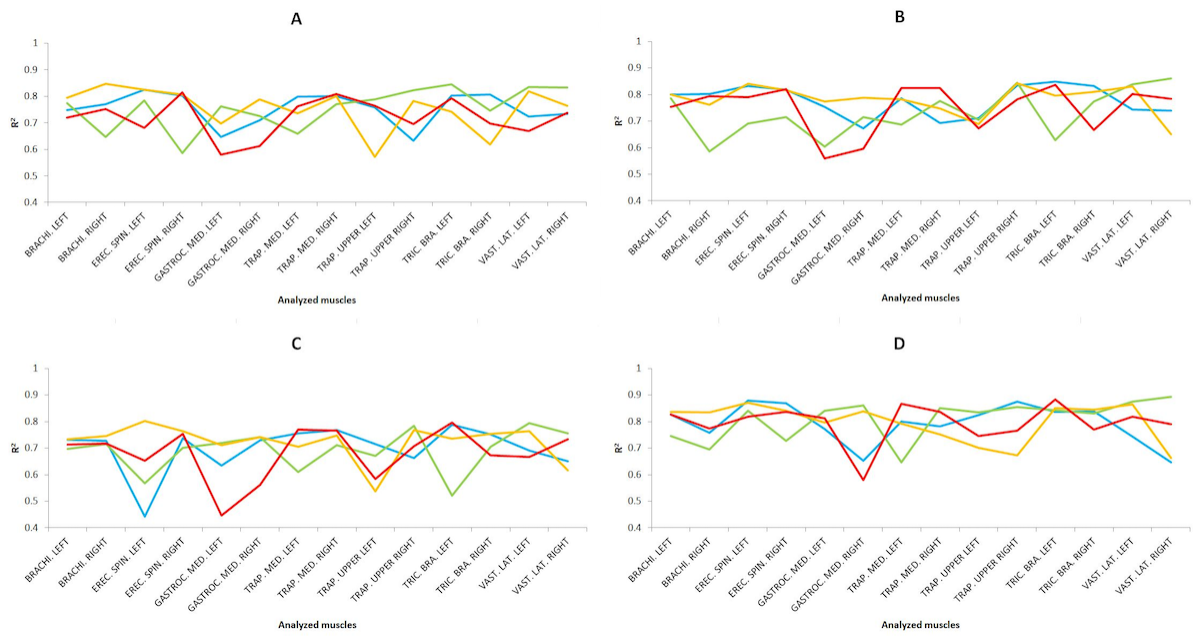
Results from the test dataset indicate the R2 values applying the best model in each case on four simulator tasks: (A) dissection, (B) labyrinth, (C) peg transfer, and (D) suturing. For the 4 analyzed groups as a function of the surgical type and the surgeons’ level of expertise, being novice surgeons and conventional laparoscopic surgery (yellow), novice surgeons and robotic-assisted surgery (red), experienced surgeons and conventional laparoscopic surgery (blue), and experienced surgeons and robotic-assisted surgery (green).

To validate the predictive models, the predicted results for the test dataset were analyzed and are shown in Figure 10 for each task. As in the previous sections, Figure 10 shows the *R*^2^ coefficient values of the test dataset for the predictive analysis performed by applying MLR and MLP, showing the higher of the two in Figure 10, with the dataset preprocessed using the scaling technique. Overall, MLR showed slightly higher values than MLP for the *R*^2^ coefficient with a low RMSE error (RMSE<0.05) for both cases.

As in the previous cases, in general, the results obtained are adequate according to the standards given by Colton [36]. We should highlight the *R*^2^ coefficient values for LAP of the right erector spinae (*R*^2^=0.8782), the left erector spinae (*R*^2^=0.8681), and the left upper trapezius (*R*^2^=0.8751) in the suturing task for expert surgeons. As for RAS, we will highlight the right medial gastrocnemius (*R*^2^=0.8599), the right middle trapezius (*R*^2^=0.8502), the left vastus lateralis (*R*^2^=0.8751) and the right vastus lateralis (*R*^2^=0.8933) and labyrinth (*R*^2^=0.8604) in the suturing task for expert surgeons, as well as the left middle trapezius (*R*^2^=0.8671), the left triceps brachii (*R*^2^=0.8837) in the suturing task for novice surgeons.

## Discussion

### Principal Findings

The muscles analyzed in this study have been extensively evaluated in previous studies [19,39] for LAP and RAS, with the main muscles analyzed being the upper trapezius, middle trapezius, and triceps brachii.

Considering the type of surgical technique, the expert surgeons had less muscle activity with the RAS technique for the dissection, peg transfer, and suturing tasks, mainly for the forearm muscles (triceps brachii, middle trapezius, and upper trapezius). However, the labyrinth task had less muscle activity with the LAP technique for the nondominant hand. Conversely, the dominant hand showed less muscle activity in the RAS technique. For the novice surgeons, the middle trapezius bilaterally and the right upper trapezius had less muscle activity in LAP for the dissection, labyrinth, and pin transfer tasks. For the other cases, they had less muscle activity using the RAS technique. In all cases, the muscle activity was lower using the RAS technique during the suturing task. The results obtained during this study agree with those obtained in previous studies [19,40], which demonstrated that from an ergonomic point of view, RAS is more comfortable than LAP [41].

In the same way, muscle activity is closely related to muscle fatigue, and prolonged muscle activity of small, single muscle fibers can cause degenerative muscle changes even at very low levels of muscle activity. For this reason, proper ergonomics is very important for LAP activities [42]. This fact reinforces the need for future surgeons in surgical robotics to receive structured, progressive, and adequate training in ergonomic aspects in both LAP and RAS [43].

Joint analysis of spectrum and analysis graphs has been widely applied in scientific literature for the analysis of muscle fatigue [44]. This method relates muscle activity and the mean frequency of the EMG spectrum, obtaining a schematic representation of the relationship between localized muscle fatigue and force [45]. Previous studies have studied trapezius muscle fatigue during LAP, as the trapezius is frequently vulnerable to localized muscle fatigue [46]. This fact is being corrected with RAS, as the activity of the main dominant muscles in LAP has been reduced [47]. Furthermore, these results showed less localized muscle fatigue and greater use of force in RAS than in LAP in novice surgeons. However, in the case of expert surgeons, the use of force and localized muscle fatigue was lower in LAP. This fact could be related to their previous experience in LAP [43]. However, it would be necessary to take into account other aspects that may affect surgeons’ ergonomics, such as the ergonomically optimal configuration of the working environment, their prior training in the field of surgical ergonomics, activities performed prior to the study, or the use of simulators of similar surgical environments, among others.

In general, all muscles bilaterally show statistically significant differences between the LAP technique and RAS. This fact demonstrates the different use of muscles between LAP and RAS for both types of surgeons, experts and novices [39]. Similarly, obtaining better results by applying MLR as an AI technique could be related to the limitations of using MLP, such as its black box nature or the need to use a large amount of data for training.

Considering more specifically the results for the tasks performed by the LAP technique (Figure 6), for the dissection task, the left brachioradialis showed lower muscle activity for novice surgeons, and the right medial gastrocnemius showed lower muscle activity for expert surgeons. For the peg transfer task, the muscles that did not follow the general trend were the medial gastrocnemius bilaterally, showing less muscle activity for novice surgeons, and the right vastus lateralis, also showing less muscle activity for novice surgeons. In the same way, the results obtained for the suturing task followed the general trend, except for the medial gastrocnemius bilaterally, displaying less muscle activity for novice surgeons, highlighting the similarity for the middle trapezius bilaterally.

In the case of the tasks using RAS (Figure 6), the dissection and labyrinth tasks followed the general trend, with less muscle activity being shown by novice surgeons, except for the right erector spinae, which showed less muscle activity for the expert surgeons. For the peg transfer task, the general trend was followed, with less muscle activity for novice surgeons, except for the erector spinae bilaterally and right middle trapezius, which displayed less muscle activity for expert surgeons. Finally, for the suturing task, the general trend was followed, with less muscle activity for novice surgeons, except for the erector spinae bilaterally and the right middle trapezius and right upper trapezius, which displayed less muscle activity for expert surgeons.

It is noteworthy that, in general, muscle activity on the right side is lower than on the left side, both for experienced and novice surgeons. This fact could be related to the dominant side of surgeons, who require less effort to complete a manual task [48]. Consistent with this fact, muscle activity on the dominant side also appears to increase slightly over time compared to the nondominant side, suggesting that the dominant side may be more susceptible to developing muscle fatigue and possible future musculoskeletal disorders [49]. In the same way, the results obtained for Figure 7 are consistent with those obtained in previous analyses [50].

The results from Figure 8 could be related to the variability of the surgeon’s surgical experience and the stress generated during the performance of surgical activities [51]. Moreover, in almost all cases, a high to excellent relationship was achieved (*R*^2^>0.75), highlighting that for the dissection task, expert surgeons with RAS achieved the highest *R*^2^ values for most parameters for the labyrinth task, expert surgeons with LAP, for the peg transfer task, novice surgeons with LAP and for the suturing task, expert surgeons with RAS. These positive predictive results are in agreement with previous studies that also showed high to excellent predictive results performing LAP and RAS [5]. As for the previous results, the predictive results for Figure 9 are in agreement with previous studies [5].

As in previous sections, these satisfactory predictive results for Figure 10 are in agreement with previous studies [5]. Moreover, in almost all cases, a good to excellent relationship was achieved (*R*^2^>0.5), highlighting that for the dissection task, expert surgeons with RAS obtained the highest *R*^2^ values for most parameters, for the labyrinth and pin transfer tasks, novice surgeons with LAP, and for the suturing task, expert surgeons with RAS. In general, the outcomes of this study exhibit a slight superiority over those obtained in preceding studies that used AI models for localized muscle fatigue prediction [52-55].

Among the limitations of the present study is the small number of participating surgeons. Although previous studies have established the relationship between muscle activity and localized muscle fatigue experienced during simulated tasks compared to surgical procedures [19], further research in this area is warranted. This should involve increasing the number of surgeons involved and conducting more intricate surgical procedures that closely resemble real-world scenarios, obtaining more definitive and representative results [56]. On the other hand, the limitations due to the number of EMG sensors available and that can be used simultaneously have prevented us from recording the muscle activity of all the muscle groups of interest. For future work, we intend to include recordings of other muscle groups, such as the deltoid or biceps brachii, which have also been shown to be related to LAP activity. Furthermore, the incorporation of additional wearable and physiological sensors, such as EDA or an electrocardiogram, should be considered in subsequent studies to enhance the representativeness of the results.

For future research, the predictive models derived from this study are intended to be integrated and validated within surgical training activities and in actual surgical practice across diverse specialties, including general surgery, urology, and gynecology, among others. This integration will contribute to the prevention of musculoskeletal issues during surgical practice and, consequently, enhance the quality of surgical outcomes. Furthermore, it would be beneficial for future studies to include participating surgeons with prior experience in RAS and to analyze the impact of experience on the ergonomics of surgeons who perform robotic surgery. Additionally, it would be insightful to investigate the correlation between the onset of localized muscle fatigue and the quality of surgical performance during RAS surgery.

Similarly, the development of real-time feedback systems would allow surgeons to be alerted to situations of high localized muscle fatigue and prevent possible musculoskeletal risks during surgical practice. In this way, predictive models could be integrated into surgical training and actual clinical practice, improving the health of the surgical team and, consequently, the quality of patient care.

### Conclusions

In this study, a novel predictive model based on MLR and MLP has been developed to predict the localized muscle fatigue of a set of surgeons’ muscle groups during LAP and RAS. This model achieved satisfactory *R*^2^ and RMSE coefficients for all parameters extracted from the EMG signal, predicting with high accuracy the localized muscle fatigue values. The MLR predictive model demonstrated superior performance relative to the MLP model. Therefore, it is a promising starting point for predicting surgeons’ ergonomic risks during surgical practice in MIS.

Regarding the analysis of localized muscle fatigue, in general, RAS showed less muscle fatigue for novice surgeons compared to LAP practice, although it was higher for expert surgeons. Regarding ergonomic aspects, postural habits and motor control are different among surgeons with different surgical experience, which affects muscle activity patterns. The prevention and improvement of ergonomics in surgical practice minimizes localized muscle fatigue and forced postures, improves the health of surgeons, favors surgical performance, and consequently, contributes to improving the quality of patient care.

Ergonomic analysis makes it possible to study individual variations in the different ergonomic parameters according to the surgeons’ experience or the surgical technique used, thus allowing for improved training and surgical guidelines. Considering the rapid expansion of RAS in clinical practice, the analysis and improvement of the ergonomic conditions of surgeons and the surgical team are essential to provide optimal working conditions, ensuring the well-being of the surgical team and the quality of patient care. The training of the surgical team in the optimization of ergonomic conditions is fundamental to maximizing the benefits of MIS for both patients and surgeons.

## Abbreviations

%MVC: percentage of maximum voluntary contraction
AI: artificial intelligence
EDA: electrodermal activity
EMG: electromyography
LAP: conventional laparoscopic surgery
MIS: minimally invasive surgery
MLP: multilayer perceptron
MLR: multiple linear regression
RAS: robotic-assisted surgery
RMSE: root-mean-square error
WEKA: Waikato Environment for Knowledge Analysis

## Data Availability

The datasets generated or analyzed during this study are available from the corresponding author on reasonable request.

## References

[1] Gabrielson AT, Clifton MM, Pavlovich CP, Biles MJ, Huang M, Agnew J, Pierorazio PM, Matlaga BR, Bajic P, Schwen ZR (2021). Surgical ergonomics for urologists: a practical guide. Nat Rev Urol.

[2] Müller DT, Ahn J, Brunner S, Poggemeier J, Storms C, Reisewitz A, Schmidt T, Bruns CJ, Fuchs HF (2023). Ergonomics in robot-assisted surgery in comparison to open or conventional laparoendoscopic surgery: a narrative review. Int J Abdom Wall Hernia Surg.

[3] Hurley AM, Kennedy PJ, O’Connor L, Dinan TG, Cryan JF, Boylan G, O’Reilly BA (2015). SOS save our surgeons: stress levels reduced by robotic surgery. Gynecol Surg.

[4] Turner SR, Mormando J, Park BJ, Huang J (2020). Attitudes of robotic surgery educators and learners: challenges, advantages, tips and tricks of teaching and learning robotic surgery. J Robot Surg.

[5] Caballero D, Pérez-Salazar MJ, Sánchez-Margallo JA, Sánchez-Margallo FM (2024). Applying artificial intelligence on EDA sensor data to predict stress on minimally invasive robotic-assisted surgery. Int J Comput Assist Radiol Surg.

[6] Kaplan JR, Lee Z, Eun DD, Reese AC (2016). Complications of minimally invasive surgery and their management. Curr Urol Rep.

[7] Wilson MR, Poolton JM, Malhotra N, Ngo K, Bright E, Masters RSW (2011). Development and validation of a surgical workload measure: the surgery task load index (SURG-TLX). World J Surg.

[8] Nillahoot N, Pillai BM, Sharma B, Wilasrusmee C, Suthakorn J (2022). Interactive 3D force/torque parameter acquisition and correlation identification during primary trocar insertion in laparoscopic abdominal surgery: 5 cases. Sensors (Basel).

[9] Armijo PR, Huang C, High R, Leon M, Siu K, Oleynikov D (2019). Ergonomics of minimally invasive surgery: an analysis of muscle effort and fatigue in the operating room between laparoscopic and robotic surgery. Surg Endosc.

[10] Guzmán-García C, Sánchez-González P, Margallo JAS, Snoriguzzi N, Rabazo JC, Margallo FMS, Gómez EJ, Oropesa I (2022). Correlating personal resourcefulness and psychomotor skills: an analysis of stress, visual attention and technical metrics. Sensors (Basel).

[11] Avila-Tomás J F, Mayer-Pujadas M, Quesada-Varela V (2020). [Artificial intelligence and its applications in medicine I: introductory background to AI and robotics]. Aten Primaria.

[12] Janiesch C, Zschech P, Heinrich K (2021). Machine learning and deep learning. Electron Mark.

[13] Luo X, Kang Y, Duan S, Yan P, Song G, Zhang N, Yang S, Li J, Zhang H (2023). Machine learning-based prediction of acute kidney injury following pediatric cardiac surgery: model development and validation study. J Med Internet Res.

[14] Lee GI, Lee MR, Green I, Allaf M, Marohn MR (2017). Surgeons' physical discomfort and symptoms during robotic surgery: a comprehensive ergonomic survey study. Surg Endosc.

[15] Kim HJ, Yang JH, Chang D, Lenke LG, Pizones J, Castelein R, Watanabe K, Trobisch PD, Mundis GM, Suh SW, Suk S (2024). Assessing the reproducibility of the structured abstracts generated by chatgpt and bard compared to human-written abstracts in the field of spine surgery: comparative analysis. J Med Internet Res.

[16] Brunner S, Müller D, Krauss DT, Datta RR, Eckhoff JA, Storms C, von Reis B, Chon S, Schmidt T, Bruns CJ, Fuchs HF (2024). Cologne ergonomic measurement for robotic surgery (CEMRobSurg) using the Hugo™ RAS system. Surg Endosc.

[17] Dwyer A, Huckleby J, Kabbani M, Delano A, De Sutter M, Crawford D (2020). Ergonomic assessment of robotic general surgeons: a pilot study. J Robot Surg.

[18] Yu D, Dural C, Morrow MMB, Yang L, Collins JW, Hallbeck S, Kjellman M, Forsman M (2017). Intraoperative workload in robotic surgery assessed by wearable motion tracking sensors and questionnaires. Surg Endosc.

[19] Pérez-Salazar MJ, Caballero D, Sánchez-Margallo JA, Sánchez-Margallo FM (2024). Comparative study of ergonomics in conventional and robotic-assisted laparoscopic surgery. Sensors (Basel).

[20] Pérez-Salazar MJ, Caballero D, Sánchez-Margallo JA, Sánchez-Margallo FM (2024). Correlation study and predictive modelling of ergonomic parameters in robotic-assisted laparoscopic surgery. Sensors (Basel).

[21] Hwang M, Ichnowski J, Thananjeyan B, Seita D, Paradis S, Fer D, Low T, Goldberg K (2023). Automating surgical peg transfer: calibration with deep learning can exceed speed, accuracy, and consistency of humans. IEEE Trans Automat Sci Eng.

[22] Healey J, Picard R (2005). Detecting stress during real-world driving tasks using physiological sensors. IEEE Trans Intell Transport Syst.

[23] Shi W, Liu PX, Zheng M (2020). Cutting procedures with improved visual effects and haptic interaction for surgical simulation systems. Comput Methods Programs Biomed.

[24] Phan PT, Hoang TT, Thai MT, Low H, Davies J, Lovell NH, Do TN (2021). Smart surgical sutures using soft artificial muscles. Sci Rep.

[25] NA (2007). [Law 14/2007, 3 July, on biomedical research (BOE, 4 July 2007)]. Rev Derecho Genoma Hum.

[26] Hermens HJ, Freriks B, Disselhorst-Klug C, Rau G (2000). Development of recommendations for SEMG sensors and sensor placement procedures. J Electromyogr Kinesiol.

[27] Dufaug A, Barthod C, Goujon L, Marechal L (2020). New joint analysis of electromyography spectrum and amplitude-based methods towards real-time muscular fatigue evaluation during a simulated surgical procedure: a pilot analysis on the statistical significance. Med Eng Phys.

[28] Oka M (2021). Interpreting a standardized and normalized measure of neighborhood socioeconomic status for a better understanding of health differences. Arch Public Health.

[29] Dietterich TG (1998). Approximate statistical tests for comparing supervised classification learning algorithms. Neural Comput.

[30] Frank E, Hall MA, Witten IH (2016). The weka workbench. Online Appendix for Data Mining: Practical Machine Learning Tools and Techniques.

[31] Fayyad U, Piatetsky‐Shapiro G, Smyth P (1996). From data mining to knowledge discovery in databases. AI Mag.

[32] Caballero D, Caro A, Dahl AB, ErsbØll BK, Amigo JM, Pérez-Palacios T, Antequera T (2018). Comparison of different image analysis algorithms on MRI to predict physico-chemical and sensory attributes of loin. Chemom Intell Lab Syst.

[33] Wu X, Kumar V, Ross Quinlan J, Ghosh J, Yang Q, Motoda H, McLachlan GJ, Ng A, Liu B, Yu PS, Zhou Z, Steinbach M, Hand DJ, Steinberg D (2007). Top 10 algorithms in data mining. Knowl Inf Syst.

[34] Bishop CM (2006). Pattern Recognition and Machine Learning.

[35] Grossman R, Seni G, Elder J (2010). Ensemble Methods in Data Mining: Improving Accuracy Through Combining Predictions.

[36] Colton T (1974). Statistics in Medicine.

[37] Hyndman RJ, Koehler AB (2006). Another look at measures of forecast accuracy. Int J Forecast.

[38] Porta-Serra M, Plasencia A, Sanz F (1988). La calidad de la información clínica: estadistícamente significativo o clínicamente importante? [The quality of clinical information: statistically significant or clinically important?]. Med Clin (Barcelona).

[39] Jarc AM, Curet MJ (2017). Viewpoint matters: objective performance metrics for surgeon endoscope control during robot-assisted surgery. Surg Endosc.

[40] Pérez-Duarte F J, Lucas-Hernández M, Matos-Azevedo A, Sánchez-Margallo J A, Díaz-Güemes I, Sánchez-Margallo F M (2014). Objective analysis of surgeons' ergonomy during laparoendoscopic single-site surgery through the use of surface electromyography and a motion capture data glove. Surg Endosc.

[41] Willuth E, Hardon SF, Lang F, Haney CM, Felinska EA, Kowalewski KF, Müller-Stich B P, Horeman T, Nickel F (2022). Robotic-assisted cholecystectomy is superior to laparoscopic cholecystectomy in the initial training for surgical novices in an ex vivo porcine model: a randomized crossover study. Surg Endosc.

[42] Wee IJY, Kuo L, Ngu JC (2020). A systematic review of the true benefit of robotic surgery: ergonomics. Int J Med Robot.

[43] Sjøgaard G, Lundberg U, Kadefors R (2000). The role of muscle activity and mental load in the development of pain and degenerative processes at the muscle cell level during computer work. Eur J Appl Physiol.

[44] Straker L, Mathiassen SE (2009). Increased physical work loads in modern work--a necessity for better health and performance?. Ergonomics.

[45] Mastaglia FL (2012). The relationship between muscle pain and fatigue. Neuromuscul Disord.

[46] Luttmann A, Jäger M, Laurig W (2000). Electromyographical indication of muscular fatigue in occupational field studies. Int J Ind Ergon.

[47] Nordander C, Hansson G, Ohlsson K, Arvidsson I, Balogh I, Strömberg Ulf, Rittner R, Skerfving S (2016). Exposure-response relationships for work-related neck and shoulder musculoskeletal disorders--analyses of pooled uniform data sets. Appl Ergon.

[48] Krzysztofik M, Jarosz J, Matykiewicz P, Wilk M, Bialas M, Zajac A, Golas A (2021). A comparison of muscle activity of the dominant and non-dominant side of the body during low versus high loaded bench press exercise performed to muscular failure. J Electromyogr Kinesiol.

[49] Luger T, Bonsch R, Seibt R, Krämer B, Rieger MA, Steinhilber B (2023). Intraoperative active and passive breaks during minimally invasive surgery influence upper extremity physical strain and physical stress response-a controlled, randomized cross-over, laboratory trial. Surg Endosc.

[50] Diederichsen LP, Nørregaard J, Dyhre-Poulsen P, Winther A, Tufekovic G, Bandholm T, Rasmussen LR, Krogsgaard M (2007). The effect of handedness on electromyographic activity of human shoulder muscles during movement. J Electromyogr Kinesiol.

[51] Rodrigues Armijo P, Huang C, Carlson T, Oleynikov D, Siu K (2020). Ergonomics analysis for subjective and objective fatigue between laparoscopic and robotic surgical skills practice among surgeons. Surg Innov.

[52] Keshavarz Panahi A, Cho S (2016). Prediction of muscle fatigue during minimally invasive surgery using recurrence quantification analysis. Minim Invasive Surg.

[53] Al-Mulla MR, Sepulveda F, Colley M (2011). A review of non-invasive techniques to detect and predict localised muscle fatigue. Sensors (Basel).

[54] Lambay A, Liu Y, Morgan PL, Ji Z (2024). Machine learning assisted human fatigue detection, monitoring, and recovery: a review. Digit Eng.

[55] Wei K, Kimura C, Shimura M, Shimomura Y, Zhao X, Tamura T, Sakamoto S (2025). Predicting task performance in robot-assisted surgery using physiological stress and subjective workload: a case study with interpretable machine learning. Front Hum Neurosci.

[56] Amirthanayagam A, Zecca M, Barber S, Singh B, Moss EL (2023). Impact of minimally invasive surgery on surgeon health (ISSUE) study: protocol of a single-arm observational study conducted in the live surgery setting. BMJ Open.

